# Supplementary material for: Genetic transformation of Gardnerella species and characterization of vaginolysin and sialidase mutants
Source: Infect Immun. 2025 Aug 21;93(10):e00299-25. doi: 10.1128/iai.00299-25 (PMC12519793; doi:10.1128/iai.00299-25)
Supplement: Supplemental tables — Tables S1 and S2. [file iai.00299-25-s0001.pdf]

Supplemental Material

Genetic transformation of *Gardnerella* species and characterization of vaginolysin and sialidase mutants

Amy K. Klimowicz[1], Erin M. Garcia[1], Kimberly K. Jefferson[2], Joseph P. Dillard[1*]

[1] Department of Medical Microbiology and Immunology, University of Wisconsin-Madison, Madison, WI, United States

[2] Department of Microbiology and Immunology, School of Medicine, Virginia Commonwealth University, Richmond, VA, USA

[*]Send correspondence to jpdillard@wisc.edu

Table S1. Bacterial strains and plasmids used in this study.

Strain or plasmid	Characteristics	Source/reference
**Bacterial strains**		
Gardnerella spp.		
ATCC 14018	*G. vaginalis* $LacZ^+$, hemolytic, $sialidase^-$	ATCC
3336	*G. pickettii* $LacZ^-$, hemolytic, $sialidase^+$, Tet^R	M. Roberts
AKK107	14018 x pAKK134 (‘*vly*’) ID* mutant, Erm^R	This work
AKK110	14018 x pAKK139 (‘*haeIII*’) ID mutant, Erm^R	This work
AKK112	14018 x pAKK144 (0.2 kb ‘*lacZ*’) ID mutant, Erm^R	This work
AKK113	14018 x pAKK145 (0.5 kb ‘*lacZ*’) ID mutant, Erm^R	This work
AKK114	14018 x pAKK146 (1 kb ‘*lacZ*’) ID mutant, Erm^R	This work
AKK115	14018 x pAKK148 (1.8 kb ‘*lacZ*’) ID mutant, Erm^R	This work
AKK123	14018 x pAKK165 (*vlymut* $pheS_{mut2}$) integrant, Erm^R 4CP-sensitive	This work
AKK124	4CP-resistant Erm^S AKK123, *vly* point mutant	This work
AKK131	14018 x pAKK176 (Δ*vly* $pheS_{mut2\_alt}$) integrant	This work
AKK132	4CP-resistant AKK131, Δ*vly* mutant	This work
AKK136	AKK132 x pAKK187 (Δ*vly*/*vly*+) complemented mutant	This work
AKK140	3336 x pAKK191 (Δ*nanH3* $pheS_{mut2\_alt}$) integrant, Erm^R	This work
AKK141	4CP-resistant AKK141, Δ*nanH3* mutant	This work
E. coli		
TAM1	Used for cloning	RapidTrans™
TOP10	Used for cloning	Invitrogen™
**Plasmids**		
pIDN1	*E. coli* cloning vector, Erm^R	(*1*)
pSPUC	*E. coli* cloning vector, source of spectinomycin resistance gene, Spc^R	(*2*)
pAKK134	0.5 kb ‘*vly*’ in pIDN1, Erm^R	This work
pAKK139	0.45 kb ’haeIII’ in pIDN1, Erm^R	This work
pAKK144	0.2-kb ‘*lacZ*’ in pIDN1, Erm^R	This work
pAKK145	0.5-kb‘*lacZ*’ in pIDN1, Erm^R	This work
pAKK146	1-kb *lacZ*’ in pIDN1, Erm^R	This work
pAKK148	1.8-kb ‘*lacZ*’ in pIDN1, Erm^R	This work
pAKK163	counter selectable construct 1; $pheS_{mut2}$ gblock in pIDN1, Erm^R	This work
pAKK165	’*vlymut*’ (point mutation) gblock cloned into pAKK163, Erm^R	This work
pAKK174	counter selectable construct 2; pheSmut2_alt gblock cloned into pIDN1, Erm^R	This work
pAKK176	Δ*vly* gblock cloned into pAKK174, Erm^R	This work
pAKK182	Spec cassette cloned into XhoI/NsiI sites of pIDN1 (replacing *ermC*), Spc^R	This work
pAKK185	‘*pknB*_‘*srtE* gblock cloned into pAKK182, Spc^R	This work
pAKK186	complementation plasmid for *G. vaginalis*; *ermC* cloned into pAKK185, Spc^R Erm^R	This work
pAKK187	*vly*+ cloned into pAKK186, Spc^R Erm^R	This work
pAKK191	Δ*nanH3* from 3336 cloned into pAKK174, Erm^R	This work

*ID = insertion-duplication mutant

Table S2. Sequences of primers, oligos and gblocks used in this study*.

Primers	Sequence 5'-3'
vly_SacI-F	CTCTGAGCTCCTCAAATTGTGAACTTCAAGC
Gv_abc-R	TGAACGCTCAACACTTGC
vly-F	CATATGAAGAGTACAAAGTTCTACC
pmob-R	AACAGCTATGACCATGATTACGCCAAG
vly_screen-F	CGTCAAGGATAACGAAGTAGC
vly_screen-R	GCTACTTCGTTATCCTTGACG
Gv_haeIII_SacI-F	GATAGAGCTCGGATCTAACTGGCAAGAGA
Gv_haeIII_ClaI-R	GCTCATCGATCAAAGGTCTGTCACCATC
haeIII_screen-F	AGGCATTGGACTGCAAACTGG
Gv_lacZ_SacI-F	CTATGAGCTCGCATCATGGCTAGAGGATC
Gv_lacZ(0.5kb)_XhoI-R	TGATCTCGAGCACATCGGAAACCATGTCA
Gv_lacZ(1kb)_XhoI-R	GTATCTCGAGTCTGGGTTGTTTGACACG
Gv_lacZ-R3	TGTAGCGTATTGTCGTCG
spec_ClaI-F	AGATATCGATTAACGTGACTGGCAAGAG
spec_NsiI-R	GACAATGCATACAGCTATGACCATGATTACG
vly_SpeI-F	CTATACTAGTCTGTAACTTCCGCAGTGA
vly_mid-F	TTCGATGCAGTACACAAGG
vly_up_screen-F	TGTGTGATGCGTGACCTTC
pknB_screen2-F	GCTGGAGAAGTTGTAGTTCC
srtE_screen3-R	GACCAGAACAGGTTGAGG
nanH3_up_SacI-F	CATAGAGCTCAGCACGCAGTAAATAGACC
nanH3_up_SpeI-R	GCATACTAGTCGCTGTTCCAATCATTATATTCC
3336nanH3_down_SpeI-F	CAGTACTAGTGGAATCAACACAACTATTCCTTATGC
3336nanH3_down_XhoI-R	TTAACTCGAGAACTAAGATGCCCTCGGT
**Oligos**	
rpsL2mut	CGTGTGTACACCACCACTCCT**CG**GAAGCCAAACTCTGCGCTTCG
rpsL4mut	CGTGTGTACACCACCACTCC**ACGC**AAGCCAAACTCTGCGCTTCG
rpsL2mut_long	CGTGGCGTGTGCACTCGTGTGTACACCACCACTCCT**CG**GAAGCCAAACTCTGCGCTTCGTAAGGTTGCTCGTGTGCGCCTCAGC
rpsL4mut_long	CGTGGCGTGTGCACTCGTGTGTACACCACCACTCC**ACGC**AAGCCAAACTCTGCGCTTCGTAAGGTTGCTCGTGTGCGC CTCAGC
**gblocks**	
0.2 kb 'lacZ' gblock	CTATGAGCTCGCATCATGGCTAGAGGATCAAGATTATTGGAGACTACACGGCATTTTCCGTTCCGTTGAACTGTGTGCACATCCTTCCACCCATGTATCAAATCTTCATGTTGATGCAGATTATAGCAATGATACAAATACTGGGAAACTCGCATTCAGAGCAAACATTGAAGGAGACAATTTAAAAGATATTACACTACACGCTTACATCTCGAGGTAT
pheSmut2 gblock	CTGAATCGATCGTAATGCGTCGTACATACCGCACATTAAAATGAACATGTTAGTTTAAATAACGAAGACCAAAACGTATTGTTAAAA

	ATCGCGAAAAATCGCTAGAACTATGCGTTAAGGTTTCACTCAA<br>GGGAGATCCCATATGCTTCACAAAAGTACACATAACACGGATA<br>GGAAGGGTGCTGTGGCAAGTAGTAAGCCGTTCGACGCGAAG<br>GCAATTACAAAGGTAGTAAAAGAGGGTATAGCTTGTGTTCAAG<br>CCGCTAAAACAATGGAAGAGTTGAAAGCGGCAAAAACTAAGT<br>ATGCAGGAGCTCAGTCTGCAATGACCCTTGCCAGCAAGTCTA<br>TTGGAAGTCTTCAAGTAGAAGAAAAGAAAGAAGCTGGCAAGA<br>TAATGTCGGCTTTACGCGCTGATTTTGGTCGTGAATTTGCAGC<br>AGCCCAAGAGCGTATTAAGGCTATTGAAGAAGCTAATATGCTT<br>CAAAAAGAAACAGTCGATATGACCTTGCCAATAAATCGTAAGC<br>CGCTTGGCGCTCGCCATCCGATTGCGCGTATTATTGAAGACT<br>TTGAAGACTTCTTTGTGTCTATGGGTTGGCAGATTTCTGCAGG<br>ACCAGAGGTAGAAACAGAGTGGTTCGATTTTGACGCGTTGAA<br>TTTTGGTCCAGATCATCCTGCTCGTCAAATGCAAGATACTTTT<br>TATGTGCAAGGTAATCAGGCAAAGGATGCTGCAGGATTTGTT<br>GGATCTAACATGGTTTTGCGTACGCAAACTTCTTCCGATCAGG<br>TTCGTGCGTTAATTGAGCGAGGAGTACCTCTGTATATTGCGTC<br>CCCAGGCCGAGTATTCCGCACGGATGAGCTTGATGCAACGCA<br>TACTCCAGTCTTCCACCAGTGCGAAGCTTTGGCAGTAGATAAA<br>CATTTGAGCATGGCTGATTTAAAGGGTGTTCTTGATCGCCTTG<br>CTGTTGCAATGTTTGGTCCAGATGCAAAAAGCCGTTTGCGCC<br>CAAGCTACTTCCCGTTC**T**C**T**GAGCCTAGTGCGGAGTTGGATC<br>TTTGGTTCCCAGATAAGAAGGGTGGCCCAGGTTGGATTGAAT<br>GGGGCGGATGCGGAATGGTGAATCCGAATGTTCTTAAGTCTG<br>CAGGCTTAGACCCAGATGTTTATACGGGATTTG**GA**TTCGGTG<br>TTGGCTTGGAGCGCACTTTGCTGCTTCGTCACGATATTAACGA<br>TATGCACGATTTGGTTGAAGGCGATAAGCGCTTCAGTGAACA<br>GTTTGTGATGGGAGAGTAGCTCGAGTATG
vlymut gblock	CTGTACTAGTGAACCAGCTACATCTTGCGCAGCTAAGAAAGA<br>CTCGTTGAATAATTATTTGTGGGATTTGCAATACGATAAAACAA<br>ACATTCTCGCCCGTCATGGCGAAACCATTGAGAACAAATTCTC<br>CAGCGACAGCTTCAACAAGAACGGTGAATTCGTTGTTGTTGA<br>GCATCAGAAGAAGAACATCACCAATACAACTTCAAATTTGTCG<br>GTTACTTCCGCCAACGATGATCGCGTATACCCAGGTGCTCTTT<br>TCCGTGCTGATAAGAATTTGATGGACAATATGCCAAGCCTGAT<br>TTCTGCAAACCGCGCTCCAATAACGTTGAGCGTTGATTTGCC<br>GGGATTCCACGGCGG**T**AAAGTGCTGTAACTGTTCAGCGCCCA<br>ACCAAGAGTTCTGTAACTTCCGCAGTGAACGGCTTAGTTTCTA<br>AGTGGAATGCACAATATGGAGCAAGTCATCATGTTGCAGCTC<br>GCATGCAGTACGATTCTGCAAGCGCACAAAGCATGAACCAGC<br>TCAAGGCTAAGTTTGGTGCTGATTTTGCCAAGATTGGTGTTCC<br>GCTGAAGATTGATTTCGATGCAGTACACAAGGGTGAGAAGCA<br>GACTCAAATTGTGAACTTCAAGCAAACTTACTACACCGTAAGC<br>GTTGATGCACCAGATAGCCCAGCAGATTTCTTTGCTCCTTGCA<br>CTACGCCAGACAGCTTGAAGAACCGTATCGATTGAG
pheSmut2_alt gblock	GTTACTCGAGCGTAATGCGTCGTACATACCGCACATTAAAATG<br>AACATGTTAGTTTAAATAACGAAGACCAAAACGTATTGTTAAAA<br>ATCGCGAAAAATCGCTAGAACTATGCGTTAAGGTTTCACTCAA<br>GGGAGATCCCATATGTTGCATAAATCTACGCACAACACTGAC<br>CGCAAAGGAGCCGTGGCGTCTAGCAAGCCGTTCGACGCTAA<br>AGCTATTACTAAAGTTGTGAAAGAAGGTATAGCCTGCGTACAG<br>GCAGCTAAAACAATGGAAGAGTTGAAGGCGGCTAAGACGAAG<br>TATGCCGGCGCTCAATCCGCTATGACACTTGCAAGCAAAAGC

	ATTGGATCCTTGCAAGTTGAGGAGAAGAAGGAAGCTGGTAAG<br>ATTATGTCTGCTTTGCGCGCGGATTTTGGCCGCGAGTTCGCT<br>GCAGCCCAAGAGCGCATTAAAGCGATTGAAGAGGCTAATATG<br>CTTCAAAAGGAAACGGTAGATATGACATTGCCGATAAACCGC<br>AAACCGCTTGGAGCACGTCATCCGATTGCGCGTATTATTGAA<br>GATTTCGAGGACTTCTTCGTGTCTATGGGATGGCAAATTTCTG<br>CGGGTCCGGAAGTTGAGACGGAGTGGTTTGATTTCGACGCGT<br>TGAATTTCGGTCCAGACCACCCAGCTCGTCAGATGCAGGATA<br>CCTTCTACGTACAAGGAAATCAGGCCAAAGATGCGGCTGGAT<br>TTGTTGGATCGAATATGGTGCTTCGTACACAGACGTCTTCTGA<br>TCAAGTTCGTGCATTGATTGAACGTGGTGTTCCACTTTATATT<br>GCGAGCCCGGGCAGGGTGTTCAGGACTGACGAACTTGACGC<br>GACTCACACCCCAGTGTTTCATCAATGCGAGGCGCTTGCTGT<br>GGATAAGCATCTTTCCATGGCCGACCTTAAAGGCGTTCTTGAT<br>AGGCTTGCTGTTGCAATGTTCGGCCCAGACGCGAAGTCTCGT<br>CTGCGCCCGTCTTACTTTCCGTTTA**GC**GAACCGTCTGCGGAA<br>TTGGACCTTTGGTTTCCGGACAAAAAAGGAGGACCAGGCTGG<br>ATAGAATGGGGTGGATGCGGCATGGTGAACCCAAATGTACTT<br>AAATCCGCTGGTCTTGACCCAGACGTTTACACGGGATTTG**GT**<br>TTTGGTGTCGGTCTTGAACGCACACTGTTGCTGAGGCATGAC<br>ATTAACGATATGCATGACTTGGTAGAGGGCGACAAACGTTTCT<br>CTGAGCAGTTTGTTATGGGTGAGTAGGGTACCTCAG
Δ*vly* gblock	GTCGACTAGTCCAGACTCACTTCTAGGAGCGCCAGAAAGCGC<br>TTCTTCTTGAGTCAATGGCGTAGCAGGCTCGTCTGGGCGCAA<br>ATACGCTTCAAGAGGTACTGGATTTGCAGTAGGTGGCACATC<br>TTCGCCAGCAACTTCCTGCACTTTGTTAATGGATTCTGCAATG<br>ACGTTTAATTCGCCTTGCAAGCGACTGATTTCTTCATCGCTTA<br>AAGCAATCTGGGACAAAACACCCAAATGCTCAATTTCTTCGCG<br>TGTGAATGTAGGCATAACCTCAACTATATGTGTGATGCGTGAC<br>CTTCTTATAGAAGAAAAAAGCAATATTGATAAAAGATTTACACA<br>CGTTACTGTAGAAATATTTTCCCAATTTTAGAACATGATTGTCG<br>ATCCATATTTTCTAGGGAAAATTATGTGTAATATTAAAAAAAAT<br>TTTATTAGTGAGACAATAAGCGAAATAACAATTATTGTCACATA<br>CAGTTTTCATAAAATTAACGTTATTTGCAGAATATTTAAATATTT<br>TAGATATCGCGCTAAATATCGCGATGCTTTTCCACAACGTGGC<br>CAATTGCATACATGACTACGCCCCAAGCAAGAACCACCAAGC<br>CTGATTGCCACCATGTAAAAACATATGCGTTTGGTGGAAGCTG<br>TGCACCCGAATTAGGGGAGCCGCCCAAGAATTTCTCCACCGC<br>TGTGGCTGGCAAAAGCTGTATAAGTATTGAATTCCACTTCGCG<br>AAATTGCTTGCAAACATAATAATGCTAAGAACACTAGGCAAAA<br>TCATCACGGCTCCAATAACGCACATAATTCCGCCAGCAGTTG<br>ACTTGCAAATCATGCCAAAGCCGTACGCCATTGCTGCTACAA<br>CAACCATAATTGCAGGAGAGCCAAGAAACAGCGTAAGAGGCA<br>ATTTCCACGCGTTGCTTCCAGATAATCCAGAAGTGTTGTCTCC<br>TATAAAGGCTAATTCTGCAGCGCCTAGCGAAACTGCCATTGC<br>CCGGGTATA
Gv pknB_srtE gblock	ATATGAGCTCGCTCTTGGTCTTATCCCAGATATTTTGGAAGAT<br>GATAAATCTTCTCAACCAGAAGGAACTTTTACAAAACAGCTTC<br>CTAAAGGTGGAGCTAAAGTTTCTTCAGGATCTAGAGTAAGCGT<br>ATGGTTCTCTGTAGGTCCACAGTCTACTAAGATTCCAGATGTT<br>ACTGGTAAATCGCAAGATGTAGCACGTAAAGCTCTTGAACGT<br>GCTGGATTTAAGATATCTAATGTTCGTGTAGAAGATAGCACGG<br>AAGTTAAGAAGAATCATGTTACTCGTACAGATCCTTCTGCAGA

	TTCGTTTGCAGACAAGGGGTCTATGGTTACCTTATACATTTCT<br>TCTGGTCTTACAAAGATCCCTGATGGTTTAGTTGGACAATCTA<br>AGGATGTTGTAACAAGCGAATTGCAAAATCTTGGTTTTACTGT<br>AAATGTTGTTGAGGAAGAATCTGATACTGCATCTGAAGGAACT<br>GTGACCAAGATGAACCCATCTTCTGGTGCTGCAGTAAAGCCT<br>CATAGCTCTGTAACAGTATATGTTTCTAAAGGAAAGCCTAAAG<br>TTGAAGTTCCTCGTTTGGCTGTTGGCACTGTAACTTTCAAGCA<br>GGCTAAACAAGTATTGGAAGCAAAAGGCTTTAAGGTTGTTGCA<br>TCTGATGCATCAGCTAAAGATGATGACATAGTTACTGGAATGT<br>CTGAAAAAGAAGGAGCAAAGATTGACAAAGGTTCAACAATCA<br>CTTTGACTGTGAAATCTGCTACTCCTCCATCGGACCTTACAAA<br>GCCAGGTACGGGATTAGATTTGAATAAACCTTCTACTGAAGCC<br>CCATCTACATCTACTACAACAACAGATACAGGTTTGTAAAAAG<br>CAAAGCATAAAGCCAGTAAGTTTAATAAGCTTACTGGCTTTCC<br>ATGGTTAGTATTAAGCGTCTGATATGATTATCTTTTGCGTGTC<br>CATTGAGCATGAATGTTGTCGCGTTATATGATTGCTCGTTTTTT<br>ATTGTCTACTCGGATACGCGAATACGCGCCATGTACGCGTCC<br>GTTCGTGTCGGTCGGCTTGGATTTAGACATATGTTATCCAAGT<br>TGCATGCGAACAGGCATGGCCAGGCAAGAACCGACACCGGT<br>CTCCACCGCGGTGGCGGCCGCACTAGTGGATCCCCCGGGCT<br>GCAGGAATTCTGTACACCTAGGATATCAAGCTTATCGATACCG<br>TCGACGGCCGGCCTTTATTATTTATTAATCTTATCTACTTACTT<br>ATTATCTACTTACCCCTTAGTAAGGCGTTGCTGTGTATGCAGA<br>CATTTCTCTTAAGAATGGTATATTTGCCGAAACAAACGGGTAT<br>ACCCATTGCATAAGTATCATAATGCATATTAGGTAGATAATTAA<br>GGAGAGAATTAATCTCACTGGAAGAATTCCAGGCTGTAGCCT<br>CATGAGTCCGCCAAAGATGCTGAATTCAGGCTTTGGCTTTAG<br>ACCTGCTTTAATCTCGCGTCTTAATGGCCACTGCCATGCAATA<br>GCTCCAGCAGCAAAGATAATCAAATAAACTATCAGCGCGCCA<br>ATCATAAGCGGAACAAGCGAATCGAGATGAGAGATCAAAGAT<br>TGTTGCTCGTTATTAACAAACTTAACTTTTCCGTTTTCATCTAA<br>AGTAGAAAGCTCTTTTGGAATACCGTCAGAAACTTTAGCCCAA<br>TAGTCAAGCTCTCCAAAGCTAACAAAGCGGAACTTAGGTGTA<br>GAGAACTTAGGTTCGCATGTAATAATGGTGATCATGCGTTTCT<br>TTGGCTCTTTACTATGGTTTATTGGATCTGGGTCAAGAACCTC<br>AACCTGTTCTGGTCTAACAATCTTGTGAGTAATGTATTTGTAAA<br>CGAACCAATAATCTTTAGTTCGAAGAATAATAGAATCTCCCTTT<br>TGGAACCTATCAACATCCGCAAGAGGCTGACCATAACCATTG<br>CGGTGACCAATAATTGTAATGTTTCCGATTTCTCCAGGCAACT<br>CAGTTTTAGGATAATGACCTAATCCTCGACGATTAAGTATCTC<br>CATATCTACACCTTCAATAACATTACGCTCCCATTGATCACCG<br>AAGCGTGGAATATAGATTCGTGCTCGAGTATC

*Restriction sites are underlined. Bolded nucleotides in oligos and gblocks represent changes to the wild-type sequence.

References

1. H. L. Hamilton, K. J. Schwartz, J. P. Dillard, Insertion-duplication mutagenesis of *Neisseria*: Use in characterization of DNA transfer genes in the gonococcal genetic island. *J Bacteriol* **183**, 4718-4726 (2001).
2. E. A. Emmert, A. K. Klimowicz, M. G. Thomas, J. Handelsman, Genetics of zwittermicin a production by *Bacillus cereus*. *Appl Environ Microbiol* **70**, 104-113 (2004).
